# Pipefish embryo oxygenation, survival, and development: egg size, male size, and temperature effects

**DOI:** 10.1093/beheco/arz101

**Published:** 2019-06-29

**Authors:** Malin Nygård, Charlotta Kvarnemo, Ingrid Ahnesjö, Ines Braga Goncalves

**Affiliations:** 1 Department of Biological and Environmental Sciences, University of Gothenburg, Gothenburg, Sweden; 2 The Linnaeus Centre for Marine Evolutionary Biology, University of Gothenburg, Gothenburg, Sweden; 3 Department of Ecology and Genetics/Animal Ecology, Uppsala University, Norbyvägen, Uppsala, Sweden; 4 School of Biological Sciences/Life Sciences, University of Bristol, Bristol, UK

**Keywords:** body condition, brood reduction, embryo density, embryo size, embryo survival, male pregnancy, male size, oxygen provisioning, Syngnathidae

## Abstract

In animals with uniparental care, the quality of care provided by one sex can deeply impact the reproductive success of both sexes. Studying variation in parental care quality within a species and which factors may affect it can, therefore, shed important light on patterns of mate choice and other reproductive decisions observed in nature. Using *Syngnathus typhle*, a pipefish species with extensive uniparental male care, with embryos developing inside a brood pouch during a lengthy pregnancy, we assessed how egg size (which correlates positively with female size), male size, and water temperature affect brooding traits that relate to male care quality, all measured on day 18, approximately 1/3, of the brooding period. We found that larger males brooded eggs at lower densities, and their embryos were heavier than those of small males independent of initial egg size. However, large males had lower embryo survival relative to small males. We found no effect of egg size or of paternal size on within-pouch oxygen levels, but oxygen levels were significantly higher in the bottom than the middle section of the pouch. Males that brooded at higher temperatures had lower pouch oxygen levels presumably because of higher embryo developmental rates, as more developed embryos consume more oxygen. Together, our results suggest that small and large males follow distinct paternal strategies: large males positively affect embryo size whereas small males favor embryo survival. As females prefer large mates, offspring size at independence may be more important to female fitness than offspring survival during development.

## Teaser

The quality of parental care provided by one parent can affect the reproductive success of both parents and therefore, choosing a good parent can be crucial during mate choice. In the broad-nosed pipefish, large males brood embryos at lower densities, that become larger but have relatively lower survival during brooding than those of small males. As females prefer large mates, our work suggests that offspring size post-birth is more important than number to female fitness.

## INTRODUCTION

Parental care is a phylogenetically widespread evolutionary strategy that can be performed by females, males or both parents (Clutton-Brock 1991). Broadly speaking, parental care increases the reproductive success of parents by improving the survival and/or quality of the offspring they care for (Clutton-Brock 1991; Kvarnemo 2010; Royle et al. 2012). Offspring care is often associated with costs to the parent providing it, such as increased energy expenditure, sometimes exacerbated by reduced feeding opportunities (DeMartini 1987), which together reduce the carer’s residual reproductive value. This significant cost results in selection to optimize the trade-off between investment in a current brood versus having resources to invest in potential future broods, in order to maximize the parent’s lifetime reproductive success (Clutton-Brock 1991; Stearns 1992; Alonso-Alvarez and Velando 2012). When parental resources are limited, an energetic trade-off between number and size of offspring can result in brood reduction, which in its broad-sense definition is simply a reduction in the number of developing embryos or young over the parental period (Mock 1994). Brood reduction is well studied in birds (Mock 2004), but it also occurs in other taxa such as fishes (e.g., Manica 2004; Sagebakken et al. 2010). Partial brood reduction is often the result of parental reallocation of resources to optimize the rearing of the remaining offspring (Parker and Mock 1987), but it can also result from sibling competition, whether encouraged by the parents or independent of them (Smiseth et al. 2007). In addition, individuals vary greatly in the parental care they provide. These differences may be intrinsic (Halupka and Borowiec 2006), the result of current body condition or life stage (e.g., senescence, Ortega et al. 2017) or of flexible allocation of resources in response to the perceived value of the current breeding event (Burley 1986; Sheldon 2000). Equally important, quality of parental care can be a sexually selected trait and used by individuals to assess the quality of potential mates (Östlund and Ahnesjö 1998; Lindström et al. 2006; Lindström and St. Mary 2008). Accordingly, the study of causes and consequences of variation in parental care quality can shed important light into patterns of mate choice and other reproductive decisions observed in nature, and it has for decades attracted the interest of behavioral ecologists and evolutionary biologists (Williams 1966; Trivers 1972; Clutton-Brock 1991; Royle et al. 2012).

When parental care is provided in fish, uniparental male care is the most common form of care (Gross and Sargent 1985; Reynolds et al. 2002). In aquatic environments, parental provisioning of oxygen to offspring is of particular importance (Jones and Reynolds 1999; Brante et al. 2003; Lissåker and Kvarnemo 2006) and fanning behavior is one of the most commonly observed parental care behaviors among fish (Blumer 1979; Keenleyside 1981; Coleman and Fischer 1991; Östlund and Ahnesjö 1998). Oxygen is important for developing embryos and juveniles in all animals, however, since oxygen has a lower diffusion coefficient and lower solubility in water than in air, it is often a limiting factor for successful development in aquatic habitats (Krogh 1941; Rombough 1988a; Lee and Strathmann 1998; Green and McCormick 2005). During ontogeny, fish embryos respire by diffusion through the surface of the egg (Rombough 1988b). In spherically shaped eggs, larger eggs have a lower surface-area-to-volume ratio than smaller eggs, and thus respiration of embryos from larger eggs has been assumed to be more constrained (Krogh 1941). Although this constraint has been argued to pose limits on the evolution of egg size in aquatic environments (Sargent et al. 1987; Rombough 1998; Hendry et al. 2001; Kolm and Ahnesjö 2005), this view has been increasingly challenged in recent years. Several studies have shown that large eggs do just as well (or better) as small eggs, even under low oxygen conditions (Einum et al. 2002; Braga Goncalves et al. 2015a,b, 2016; Polymeropoulos et al. 2016; Rollinson and Rowe 2018). This is likely to occur if and when embryo oxygen requirements increase more slowly than egg surface area with increasing egg size so that the respiratory consequences of a lower surface-area-to-volume ratio are less pronounced than previously estimated (Einum et al. 2002; Braga Goncalves et al. 2016).

In fish, larger eggs commonly develop into larger embryos and juveniles that experience faster growth and better survival (Ahnesjö 1992b; Kamler 2005; Kolm 2005; Kamler 2008). Therefore, the production of larger eggs may be selected due to the higher fitness benefits of producing larger offspring. Yet, we do not observe ever increasing egg sizes in nature. Rather, females of some species produce eggs of a range of sizes, even within a clutch (e.g., salmonids: Einum et al. 2002; clownfish: Green et al. 2006), raising important questions regarding evolutionary relationships between egg size and parental care (Einum and Fleming 2004; Kolm and Ahnesjö 2005). For instance, how does egg size affect the amount or quality of parental care needed for successful development? Or conversely, how are small and large eggs affected by parental ability?

The family Syngnathidae (pipefishes, seahorses, and seadragons) has a specialized form of parental care, with males brooding the developing embryos on their bodies from mating to birth. This is achieved by ventral attachment of the eggs directly onto the male’s body or by having a (more or less) specialized brood pouch (Wilson et al. 2001) into which the female transfers her eggs at mating. In genera with more complex brood pouches, such as *Syngnathus* and *Hippocampus*, the type, quality and amount of care provided during brooding has similarities with the mammalian pregnancy (Ripley and Foran 2009; Ripley et al. 2010) and thus brooding males of these species are described as pregnant (Stölting and Wilson 2007; Kvarnemo et al. 2011).

We worked with the broad-nosed pipefish, *Syngnathus typhle* L. 1758, a temperate pipefish species native to the Eastern Atlantic Ocean. Males of this species have 2 skin folds that form a pouch where females deposit their eggs (Wilson et al. 2001; Wilson et al. 2003). Egg size is positively correlated with female body size (Braga Goncalves et al. 2011; Mobley et al. 2011) but shows little variation within a female’s egg batch (I Braga Goncalves, unpublished data, Sogabe and Ahnesjö 2011). Females adjust the protein content of their eggs positively (i.e., reproductively compensate) when they mate with smaller partners, of presumably lower perceived quality, but they do not adjust the size of their eggs (Braga Goncalves et al. 2010). After mating, males of this genus protect the embryos, osmoregulate and provide nutritional resources beyond the yolk-sac (Kvarnemo et al. 2011) until parturition, when independent juveniles emerge from the pouch. Brood reduction occurs in broad-nosed pipefish in the sense that approximately 20% of the eggs initially transferred into the brood pouch fail to develop during the pregnancy, that is, are lost (Ahnesjö 1992b, 1996; Braga Goncalves 2010; Sagebakken et al. 2011). Since we know that male body tissues can take up nutrients of maternal origin from the pouch during brooding (Sagebakken et al. 2010), it is likely that nutrients from such lost eggs or embryos are absorbed by the fathers.

Brooding males provide an oxygenated environment to the developing embryos in the pouch (Braga Goncalves et al. 2015a). Using a fine fiber-optic probe, Braga Goncalves et al. (2015a) demonstrated the possibility to measure oxygen saturation levels in the brood pouch fluids during embryo development. Brood pouch oxygen levels were lower than in the water surrounding the brooding males and decreased over the brooding period as embryos developed (Braga Goncalves et al. 2015a). In a follow-up study, embryo survival was found to be similar for large and small eggs regardless of ambient oxygen levels, but within-pouch oxygen levels were not assessed (Braga Goncalves et al. 2015b). In addition, larger males have longer and wider brood pouches that can fit a higher number of eggs (Braga Goncalves et al. 2015c) but also potentially offer a less dense brooding environment for the developing embryos. Lower embryo densities in the pouch may be beneficial for nutrient and oxygen supply to the offspring, and so it may provide an adaptive explanation as to why females prefer to mate with large males (Berglund et al. 1986a). Here, we assess whether: 1) egg size affects oxygen saturation levels in the pouch and 2) pouch oxygen saturation levels differ between differently sized males. We investigated these questions using large and small males that were mated with either large or small females, providing large and small eggs, respectively (Braga Goncalves et al. 2011). After 18 days of brooding (i.e., about one-third of the male’s pregnancy time; Ahnesjö 1995), we measured oxygen saturation levels, egg density in the pouch, embryo mass, embryo survival, and male body condition. Warmer temperature decreases oxygen solubility in water (Krogh 1941; Weiss 1970) at the same time as it increases the metabolism, and hence oxygen consumption, of adult fish (Beamish 1964). In addition to these effects, embryo development is faster at higher temperatures (Hamor and Garside 1977; Rombough 1988a; Ahnesjö 1995; Milner et al. 2010) and oxygen consumption increases with stage of development (Hamor and Garside 1977; Rombough 1988a; Green 2004). Since temperature changed over the experimental period, this variable was also included in our analyses.

## METHODS

### Animal collection and general husbandry

Broad-nosed pipefish were caught in shallow eelgrass (*Zostera marina*) meadows on the Swedish west coast (58°15′N, 11°28′E), using a beam trawl (mesh size 4 mm) pulled behind a small boat. The experiments were conducted at the nearby Klubban biological station, Uppsala University, Fiskebäckskil, in May and June of 2016.

Collected individuals were sorted by sex and size and kept in separate 100- to 225-liter storage tanks and barrels for 2–8 days, until the start of the experiment. All tanks and barrels were provisioned a continuous flow of seawater pumped straight from the sea and artificial seagrass for the fish to hide and rest in. Throughout the experiment, all fish were fed 3 times per day with live crustaceans; mainly cultured *Artemia* occasionally supplemented with wild-caught brown shrimps (*Crangon crangon*) and mysid shrimps (Mysidae). The aquarium rooms had a natural daylight regime, that is, between 15.5 and 18 h of daylight through windows, enhanced with artificial lights on a timer (on from 5 AM to 10 PM). Water temperature was measured daily, it varied and increased naturally during the study period, from 9.0 to 17.4 °C. All tanks were cleaned at least every second day.

### Ethical note

This experiment was carried out in accordance with Swedish regulations and approved by the Ethical Committee for Animal Research in Gothenburg (Dnr 86–2013 and 34–2016).

### Experimental procedure

All pipefish had their standard body length (from tip of rostrum to end of caudal peduncle) measured to the nearest mm and were separated into 2 nonoverlapping size classes: small and large for each sex (Table 1). Female body size is positively correlated with egg size in this population (Braga Goncalves et al. 2011; Mobley et al. 2011), so the female body size classes created 2 egg-size classes (small and large). To test for effects of male size and egg size on egg density, embryo survival, embryo mass and within-pouch oxygen levels, a 2 x 2 cross design was implemented with the following 4 treatments: SS—small males brooding small eggs (*N* = 18); SL—small males brooding large eggs (*N* = 30); LS—large males brooding small eggs (*N* = 14) and LL—large males brooding large eggs (*N* = 24, Table 1).

**Table 1:** Experimental mating set-up with treatment, sample size (*N*), mean and range of male and female standard length and mean number of females available to mate with per 10 males

Treatment	*N*	Male length (mm) Mean (range)	Female length (mm) Mean (range)	Mean number of females per 10 males
Small males Small eggs (SS)	18	144.4 (109–161)	162.5 (108–185)	13.5
Small males Large eggs (SL)	30	148.5 (135–164)	219.0 (190–258)	7.5
Large males Small eggs (LS)	14	194.1 (177–207)	164.2 (125–185)	19.0
Large males Large eggs (LL)	24	193.9 (176–210)	220.1 (190–258)	11.9

Matings occurred between 16 and 25 May 2016. We used 4 mating barrels (225 liters), 1 for each of the 4 treatments. Initially, 10 males of each treatment were placed into the corresponding barrel for mating. Between 7 and 20 females were added to each barrel. Number of females differed between barrels because small males can fit fewer eggs into their brood pouches than large males, and because large females are much more fecund, that is, they produce significantly more eggs, than small females. Therefore, more small females were required to fill up the brood pouch of large males than large females to fill up the brood pouches of small males. Accordingly, males in the SL treatment had access to the fewest females per male, whereas males of the LS treatment had access to the most (Table 1). The barrels were checked every 24 h for mated males with pouches fully filled with eggs. As the skin of the pouch folds are semi-translucent and the eggs are large and colorful, assessment of pouch fullness can be done visually through the pouch. Mated males were replaced in the mating barrels by new unmated males. Similarly, females that looked slim, indicating that they had mated, were removed and replaced by new unmated females. All mated females were returned to the bay where they had been caught.

Once a male was fully mated, he was removed from the barrel and measured for: 1) standard length on a millimeter board and 2) wet weight on a digital balance, and thereafter placed into a brooding tank (30–40 liter). We kept a maximum of 4 males in each brooding tank, 1 male from each treatment, differing in length and color for individual identification. Each male brooded for 18 days. As water temperature naturally increased during the study, males that mated early in the experiment brooded at lower average temperatures than males that mated later in the experiment. To take this variation into account, we calculated for each male the mean temperature for its specific brooding period. The first male to mate experienced a mean brooding temperature of 12.7 ± 0.5 °C over the 18 days of experimental brooding and the last male to mate had a mean brooding temperature of 15.8 ± 0.3 °C. On day 18 of their brooding period, the males were removed from their tanks and their pouch oxygen levels were measured, as described below. Thereafter, the males were re-measured for standard length and wet weight. Finally, males were euthanized using an overdose of MS-222 diluted in seawater and cut in half at the anus. The tail with the embryos was preserved in ethanol (96 %) for later dissection, whereas the torso was frozen (−20 °C) so that the hepatosomatic index (HSI) could be determined at a later stage (see below).

### Pouch oxygen saturation measurements

Saturation levels in the pouch fluids were measured using a thin probe and Pyro oxygen logger V3.213 with FireStingO_2_ (Firmware 3.07, Pyro Science). To calibrate the probe, seawater was bubbled with air for several minutes to reach 100% saturation level (21% dissolved oxygen) and sodium sulfite, Na_2_SO_3_ (30 g/liter) was dissolved in seawater to attain a 0% oxygen standard. As oxygen solubility depends on water temperature, the probe was equipped with a thermometer to ensure that water temperature was measured and the percent oxygen saturation was correctly assessed. Our previous work has shown that oxygen saturation levels differ between different sections of the brood pouch (Braga Goncalves et al. 2015a). We, therefore, measured oxygen levels in 2 sections inside the pouch of each male: at the bottom (i.e., posterior section) and in the middle section of the pouch. To keep the male immobile during the oxygen measurements, the male was placed inside a silicon tube with seawater and an opening with access to the pouch (see Braga Goncalves et al. 2015a for further details). Within each treatment, we alternated which section of the pouch was measured first. During measurements, oxygen saturation was considered stable when it showed at least 5 consecutive values within one oxygen percentage. The oxygen measuring software took measurements at 1 s intervals. The overall handling time per fish was around 5 min.

### Paternal body condition

We calculated 2 condition indices that are commonly used to estimate body condition in fishes: Fulton’s condition index, which is a non-lethal method based on the relationship between body length and weight, and the HSI, which is the ratio of liver mass to body mass.

We used Fulton’s condition index (body wet weight/standard length^3^) *100, to assess changes in paternal condition over the brooding period, as estimated at the start of the pregnancy and after 18 days of brooding. From this, the relative change in condition was calculated for each male as (Fulton’s index after-Fulton’s index before)/Fulton’s index before. Because broad-nosed pipefish males carry the embryos in their brood pouch, the index necessarily includes both the male body mass and the mass of the embryos.

We used the HSI to assess the final energy status of the males. Among its many physiological functions (Marshall and Hughes 1980), the liver is an important lipid storage organ that mediates energy expenditure in costly processes such as growth and reproduction (Sopinka et al. 2009), disease resistance and survival (Schloesser and Fabrizio 2016; Sagebakken et al. 2017). We calculated the HSI at experimental termination, using only the upper half of a male’s body to ensure it was not influenced by brood mass. After defrosting, the torso was opened along the ventral side and the liver was removed. The liver and the torso were dried in a drying oven (60 °C) for ≥24 h before being weighed; the torso to the nearest 0.1 mg on an analytical balance and the liver to the nearest 0.001 mg on a Cahn electronic microbalance. These weights were then used to calculate the HSI as (liver mass/torso mass) *100.

### Egg numbers, egg density, and embryo survival

The length of the pouch and width were measured using a ruler and calipers, respectively. The brood pouch was cut open and the contents removed under a stereo microscope with 6x magnification. Total number of eggs was calculated by adding up the number of developing embryos (at the expected developmental stage based on water temperature during brooding), underdeveloped eggs and unfertilized/failed eggs, to provide an estimate of the total number of eggs initially received at mating. Using the length and width measurements of the pouch, we calculated the volume of the brood pouch, in mm^3^, as 2 half cones joined at the base, following the formula: V = 2*(π*r^2^*0.5*h*)/3 where *r* is half the width of the pouch and *h* is the length of the pouch. Egg density was calculated as total number of eggs/V. Embryo survival was calculated as (number of developing embryos/total number of eggs) *100. To get an average mass per embryo (mg) for each male, a sample of 3 to 10 developing embryos (including the yolk sac) were dried and weighed in the same way as the livers, and the embryos’ weight was divided by the number of embryos used. Embryonic development was also noted. As developmental rate depends on water temperature (Ahnesjö 1995), males that started brooding towards the end of the experiment, experienced higher temperatures and had further developed embryos on day 18. Descriptive statistics (mean ± SE) for male brood pouch dimensions, number of eggs received, egg density, number of developing embryos at day 18, embryo survival and weight are provided for each treatment in Table 2.

**Table 2:** Treatment averages (mean ± SE) of male brood pouch measurements, brooding and embryo estimates

Treatment	Pouch length (mm)	Pouch width (mm)	Eggs received	Egg density (egg/mm^3^)	Developing embryos	Relative survival (%)	Embryo weight (mg)
Small males Small eggs	48.6 ± 1.5	4.3 ± 0.1	83.8 ± 5.2	0.71 ± 0.04	64.0 ± 5.6	74.2 ± 4.2	0.84 ± 0.02
Small males Large eggs	50.1 ± 0.7	4.3 ± 0.1	74.6 ± 2.6	0.61 ± 0.02	57.3 ± 2.8	75.6 ± 3.1	1.06 ± 0.03
Large males Small eggs	66.3 ± 1.1	5.5 ± 0.2	125.1 ± 6.3	0.48 ± 0.02	77.8 ± 7.5	63.8 ± 5.5	0.99 ± 0.04
Large males Large eggs	60.1 ± 0.9	5.6 ± 0.1	118.2 ± 6.6	0.43 ± 0.02	74.8 ± 7.6	64.7 ± 5.2	1.16 ± 0.04

### Statistics

All statistical analyses were carried out in SPSS 24. To control for temperature-driven effects on embryo development, as well as potential effects of temperature on oxygen saturation, and on metabolism of offspring and fathers, we included mean water temperature for each male’s brooding period as a covariate in all tests. If the covariate did not affect the response variable significantly, it was subsequently removed from the model. Male size class (small or large) and egg size class (small or large) were used as fixed factors. Thus, we analyzed most of our data using 2-factor ANCOVAs or ANOVAs. Because within-pouch oxygen saturation levels were measured in 2 sections of the pouch in each male, oxygen levels were analyzed using a linear mixed model (LMM) that included male identity as a random factor. Residuals were tested for normality and homogeneity of variances, and when deviating, response variables were either log10-transformed (oxygen level, total number of eggs, and number of developing embryos) or squared (HSI). Three livers were lost during handling, resulting in a smaller sample size for HSI, than for the other tests. In addition, embryos of 8 males were not weighed (1 SS, 1 LS, 3 SL, and 3 LL).

## RESULTS

### Number, survival, weight and density of eggs and embryos

**Figure 1:**
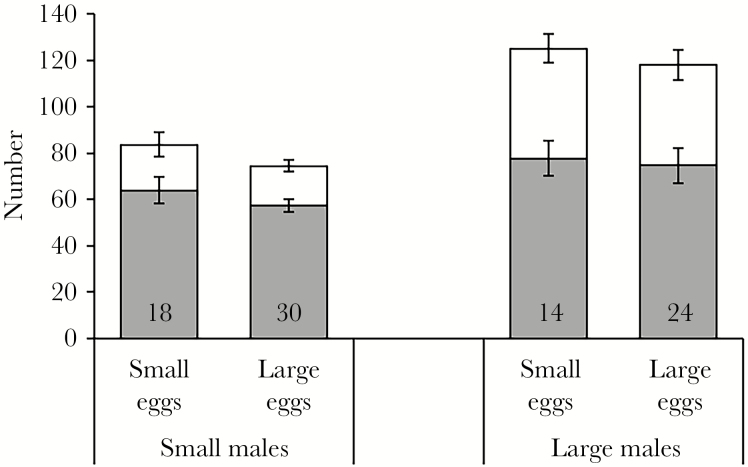
Average (mean ± SE) number of eggs initially received (white bars) and number of developing embryos (gray bars) in the brood pouch of broad-nosed pipefish males after 18 days of brooding. Sample sizes are provided at the bottom of the bars.

Large males received significantly more eggs than smaller males did (Figure 1), whereas egg size class had no effect on egg numbers and there was no interaction between male size class and egg size class (Table 3). Similarly, on day 18, large males showed a strong but nonsignificant trend towards brooding more developing embryos, whereas egg size class had no significant effect, and there was no significant interaction between the 2 factors (Table 3, Figure 1). In contrast, small males experienced significantly higher relative embryo survival, whereas again, egg size class had no impact and there was no interaction between the factors (Table 3).

**Table 3:** Effects of male size class, egg size class and their interaction on number of eggs received, number of developing embryos at day 18 of brooding, relative embryo survival, average embryo mass, and average egg density

		Male size		Egg size		Interaction	
	df	*F*	*P*	*F*	*P*	*F*	*P*
Eggs received	1,82	35.47	**<0.001**	2.01	0.160	<0.001	0.981
Developing embryos	1,81	3.92	0.051	0.36	0.553	<0.001	0.995
Embryo survival	1,82	5.84	**0.018**	0.10	0.752	<0.001	0.979
Embryo mass	1,74	14.04	**<0.001**	34.40	**<0.001**	0.46	0.499
Egg density	1,82	55.38	**<0.001**	8.56	**0.004**	0.87	0.354

The table shows the result of 5 separate 2-factor ANOVAs. Significant effects are shown in bold. Temperature was included as covariate in all 5 cases, but found nonsignificant and therefore removed from the models.

**Figure 2:**
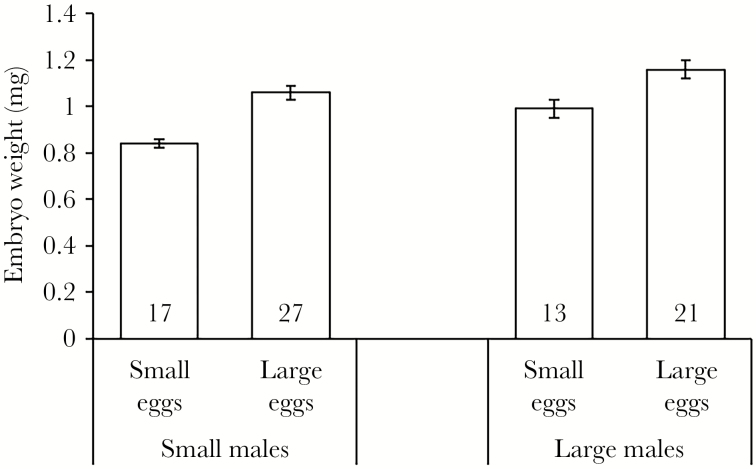
Average embryo mass (mean ± SE, mg) for small and large males of broad-nosed pipefish after 18 days of brooding either small or large eggs. Sample sizes are provided at the bottom of the bars.

Large males brooded heavier embryos for both egg size classes, and embryos from the large egg size class were significantly heavier than small ones, but there was no interaction between male size and egg size (Table 3, Figure 2).

**Figure 3:**
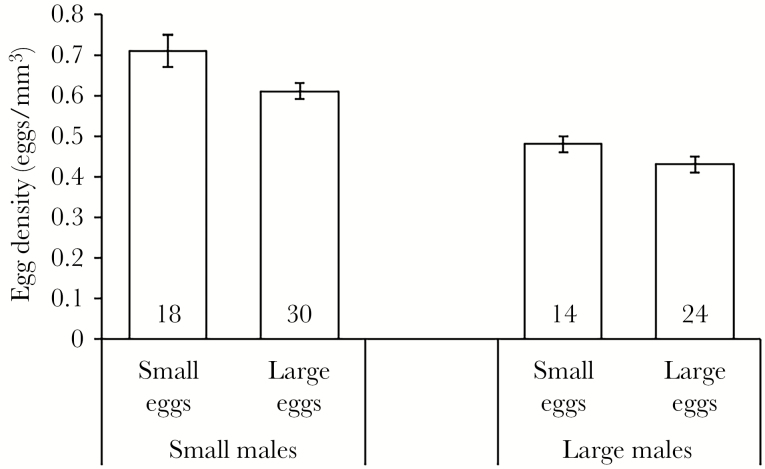
Average egg density (mean ± SE, eggs/mm^3^) in the brood pouches of small and large broad-nosed pipefish males after 18 days of brooding either small or large eggs. Sample sizes are provided at the bottom of the bars.

Average egg density in the pouch was significantly affected both by male size and egg size, but not by their interaction (Table 3). Specifically, small males had higher egg densities in their pouches independently of egg size treatment, and the small egg size class resulted in higher pouch egg densities for both small and large males (Figure 3).

### Within-pouch oxygen saturation levels

**Figure 4:**
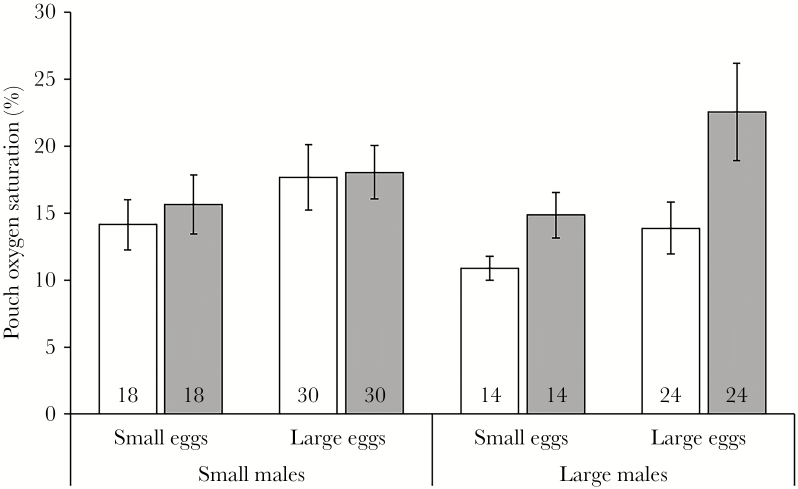
Pouch oxygen saturation levels (mean ± SE, %) in the middle (white) and bottom (gray) sections of the brood pouch, on day 18 of the brooding period, of small and large broad-nosed pipefish males brooding small or large eggs. Sample sizes are provided at the bottom of the bars.

Overall, oxygen saturation levels decreased significantly in males that had experienced higher mean brooding temperatures (LMM: temperature: *F*_1,164_ = 14.04, *P* < 0.01). Furthermore, there was a significant difference in oxygen saturation levels between the pouch sections (*F*_1,164_ = 6.89, *P* = 0.01), with saturation levels being significantly higher in the bottom section (mean: 18.3 ± 1.4% saturation) than in the middle section (mean: 14.7 ± 1.1% saturation, Figure 4). Neither male size class nor egg size class had a significant effect on within-pouch oxygen levels (male size: *F*_1,164_ = 1.37, *P* = 0.24; egg size: *F*_1,164_ = 1.04, *P* = 0.31; interaction: *F*_1,164_ = 0.05, *P* = 0.83), nor did they show a significant interaction with pouch section (male size*section: *F*_1,164_ = 3.36, *P* = 0.07; egg size*section: *F*_1,164_ = 0.12, *P* = 0.74; the trend towards an interaction between male size and pouch section relates to a larger oxygen saturation difference between the sections in large males, compared to small males; Figure 4).

### Paternal body condition

Relative change in paternal condition, measured as change in Fulton’s condition index over the brooding period, showed an average decrease in all treatments (SS: −5%, SL: −7%, LS: −3% and LL: −6%), but neither male size nor egg size had significant effects on the decrease in condition. Temperature, however, impacted paternal body condition (ANCOVA: male size: *F*_1,81_ = 1.37, *P* = 0.25; egg size: *F*_1,81_ = 0.05, *P* = 0.82; interaction: *F*_1,81_ = 0.06, *P* = 0.81; temperature: *F*_1,81_ = 19.16, *P* < 0.01), with males brooding at higher mean temperatures decreasing less in condition. At experimental termination, male HSI did not differ between treatments (male size: *F*_1,78_ = 1.72, *P* = 0.19; egg size: *F*_1,78_ = 1.31, *P* = 0.26; interaction: *F*_1,78_ = 0.39, *P* = 0.54).

## DISCUSSION

Our results demonstrate differences in parental care strategies between small and large broad-nosed pipefish males, with small males favoring embryo survival (quantity) and large males favoring embryo size (quality) during pregnancy. We also show that although egg size impacts brooding egg density and embryo size, it affects neither oxygen levels within the pouch, nor embryo survival. Despite following different parental strategies, small and large males incur similar losses in body condition. Our results, therefore, provide evidence that in the broad-nosed pipefish there is a trade-off between the size and the number of offspring males can care for and that males favor one or the other option depending on their own body size. Below we discuss these results in more detail.

### Effects of male size

Despite caring for larger absolute numbers of eggs and embryos, owing to their larger brood pouches, large males brooded them at significantly lower densities relative to small males. We also found positive effects of male size on embryo mass independent of egg size, supporting previously reported suggestions of better parental abilities in large males (Sandvik et al. 2000; Braga Goncalves et al. 2015a,b). Yet, large males had lower relative embryo survival compared to small males, meaning that initially lower brooding densities in the pouches of large males were further reduced over the course of the pregnancy. Brooding pipefish males transfer nutrients to developing embryos (Kvarnemo et al. 2011) and can take up nutrients that originate from eggs or embryos in the brood pouch (Sagebakken et al. 2010). It is, therefore, possible that large males supplied more nutrients per embryo, due to the lower egg density in their brood pouch. We speculate that large males may have attained heavier embryos by reallocating nutrients emanating from lost embryos to developing ones. That said, based on both the changes in Fulton’s index values and on the HSI estimates, large males lost similar levels of body condition during brooding and displayed similar energy status at the end of the brooding period as small males, which may suggest that the “lost” eggs were not used to minimize losses in paternal body condition.

It is common for parental investment to correlate with the size or age of the parent as found in amphibians and birds (Howard 1978; Petrie 1983), as well as in fishes, in which indeterminate growth is common (e.g., Lindström and Hellström 1993; Wiegmann and Baylis 1995). For instance, in smallmouth bass, *Micropterus dolomieu*, large males begin nesting earlier in the season (Ridgway et al. 1991), spend more time on brood defense than smaller males (Mackereth et al. 1999), and are preferred by females (Hanson and Cooke 2009). Similarly, previous studies on the broad-nosed pipefish have shown that females prefer to mate with larger partners, which can accommodate a greater number of eggs in the brood pouch and produce larger offspring (Berglund et al. 1986b; Ahnesjö 1992b). Once emerged from the brood pouch, larger juveniles have higher growth rates and better initial survival prospects (Ahnesjö 1992a,b). Our current results add to this knowledge and suggest that large males are preferred partners also because they positively affect offspring body size at emergence, independently of initial egg size. In accordance with this line of evidence, when mated with small (less preferred) males, females show reproductive compensation by transferring eggs with a significantly higher protein content than when the same female mates with a large male (Braga Goncalves et al. 2010). Given the female preference for large males (Berglund et al. 1986b), our results, therefore, indicate that, to the female, initial juvenile size is more important than relative embryo survival. To small males, however, which brood lower numbers of embryos within each pregnancy, investment into offspring number rather than size appears to be favored.

### Effects of egg size

We found few effects of egg size on the variables measured in this study. Smaller eggs resulted in more densely packed broods across both male size classes, and in lower embryo mass, which is in line with previous findings (Ahnesjö 1992a,b; Braga Goncalves et al. 2016). Egg size did not affect embryo survival nor pouch oxygen saturation levels, consistent with other studies on *S. typhle* (Mobley et al. 2011; Braga Goncalves et al. 2015b), but opposed to expectations from theory (Sargent et al. 1987; Hendry et al. 2001). Together, our results support a growing number of studies, on a variety of taxa, that have been unable to show that large eggs, with a supposedly unfavorable surface-area-to-volume ratio for gas diffusion, are more susceptible to oxygen limitations during development (Einum et al. 2002; Braga Goncalves et al. 2015b; Polymeropoulos et al. 2016; Rollinson and Rowe 2018). However, the smaller embryo sizes observed in the more densely packed broods of small males may result from constraints in other types of paternal investment, such as nutrient provisioning, as found in burying beetles (Monteith et al. 2012), or due to sibling competition, as found in newts (Vignoli et al. 2018).

### Oxygen saturation in male brood pouch: male size, egg size, and position

Overall oxygen saturation levels in the brood pouch fluid of pregnant male broad-nosed pipefish were affected neither by the size of the eggs nor by the paternal body size. Pouch oxygen levels are likely to be influenced by the respiration rate of embryos, but also by paternal oxygenation ability via blood vessels in the highly vascularized pouch tissue (Ripley et al. 2010). The lack of an egg size or male size effect could be a product of large and small eggs consuming similar amounts of oxygen during initial stages of development (standard demand), of males providing a standard oxygen environment independent of their size (standard supply), or it could be a byproduct of different oxygen demands due to number and size of developing embryos resulting in overall similar oxygen demand (net balance). However, pouch oxygenation potentially faces complex trade-offs related to embryo survival, selection for embryo size and embryo competition (as discussed below); all factors that may influence pouch oxygen saturation levels (Braga Goncalves et al. 2016).

Levels of dissolved oxygen inside the pouch were significantly higher in the bottom than the middle section, confirming previous findings in the same species (Braga Goncalves et al. 2015a), particularly so in large males. Why oxygen levels are higher in the bottom section of the pouch is not clear; it may be a result of lower local embryo density in the bottom section because the eggs are arranged in fewer rows at the bottom and are thus in closer contact with the vascularized pouch walls (personal observations). If lower egg densities result in significantly higher pouch oxygen levels, we would expect large males, which brooded embryos at lower densities, to provide a more oxygenated environment to the developing embryos than smaller males. Yet, this was not the case. Possibly, the natural temperature variation in the current study masked density effects on oxygen levels, found in a previous study (Braga Goncalves et al. 2015a), where temperature was kept constant and embryos were of more similar developmental stages when oxygen levels were measured.

### Effects of water temperature

Water temperature varied and increased over the experiment, such that males that started brooding a few days later, did so in warmer water. Males that brooded at higher average water temperature had significantly lower pouch oxygen saturation levels than males that brooded in cooler conditions. Water temperature negatively affects the amount of oxygen dissolved in the water (Krogh 1941), and positively affects fish metabolism (Beamish 1964), embryo developmental rates (Dannevig 1895; Blaxter 1969; Hamor and Garside 1977; Ahnesjö 1995) and consequently also embryo oxygen consumption (Hamor and Garside 1977; Rombough 1988a; Green 2004). In our study, late broods were more developed due to the higher temperature, and we know that embryo oxygen consumption increases with embryo development (Braga Goncalves et al. 2015a). Therefore, the lower pouch oxygen saturation levels recorded in males that brooded at higher temperatures are most likely due to a combination of these effects. These results are common and not unique to fish. For instance, in the crab *Cancer setosus* oxygen consumption at the center of the embryo mass increases with developmental stage and temperature, and oxygen consumption by brooding female crabs increases correspondingly (Brante et al. 2003).

Males brooding at lower temperature experienced a greater decrease in condition over the brooding period (i.e., change in Fulton’s index) than males brooding in warmer water. As the metabolism of both father and offspring should be higher in warmer water (Beamish 1964; Johnston and Dunn 1987), we expected embryos to be further developed and fathers to suffer greater losses in condition in warmer water. Yet, the embryos of males that brooded in warmer waters were not heavier, so their contribution towards our estimates of change in paternal condition cannot account for the smaller losses in condition in warmer water. Because Fulton’s condition index is based on overall body mass, it has been suggested to reflect changes in appetite (Thompson 1942; in Nash et al. 2006), and food intake and feeding activity have been shown to increase rapidly in some species with increases in water temperature (e.g., goldfish, *Carassius auratus*, Chen et al. 2019). In other species, lower temperatures reduce feed conversion efficiency (e.g., Atlantic salmon, *Salmo salar*, Handeland et al. 2008) and nutrient digestibility (e.g., rainbow trout, *Oncorhynchus mykiss*, Azevedo et al. 1998), both of which may negatively impact growth and body mass. If water temperature has similar effects on broad-nosed pipefish, we speculate that higher food intake in warmer water and/or reduced food digestibility and nutrient conversion efficiency in colder water, could help explain the more modest decrease in paternal condition (change in Fulton’s index) in males that brooded in warmer water, despite the predicted faster metabolic rates at higher temperatures. Still, male condition at experimental termination (HSI) was not significantly impacted by temperature. These complex effects of temperature on food intake rates, digestion efficiency and metabolism, may have led to the change in Fulton’s index, but not in our estimates of the energy status of the males (HSI). A previous study in this species did not find a relationship between feeding regime and HSI in pregnant males (Sagebakken et al. 2017). Thus, different condition indices can show disagreements if they reflect energy reserves that are stored in different tissues, used at different time scales and/or for different purposes (Schloesser and Fabrizio 2016, 2017). We, therefore, advise caution in the interpretation of the reported influence of temperature on paternal loss of condition.

### Conclusions

To conclude, large broad-nosed pipefish males showed better paternal quality in terms of giving rise to heavier embryos following 18 days of brooding, independent of initial egg size. This result provides an important explanation to why females prefer to mate with large males in this species. Yet, this greater paternal ability was not expressed via higher brood pouch oxygen levels provisioned to the embryos, although pouch oxygen levels differed significantly between sections of the pouch, particularly in large males. In contrast, small males brooded more densely packed embryos, which had better survival, but lower embryo mass, compared to larger males. Overall, our results show that, in this species, the male pregnancy involves several complex trade-offs between offspring number and size, for which optimal reproductive output appears to involve contrasting strategies for large and small males, albeit at similar energetic costs.
